# State of Health in Tobacco Manufactories

**Published:** 1844-10-19

**Authors:** 


### STATE OF HEALTH IN TOBACCO MANUFACTORIES.

M. Siméon has recently published a report on the health of the workmen employed in the manipulation of tobacco, which is not wanting in interest, and completely confirms the observations previously made on the same subject. It would appear that the fabrication of tobacco not only does not exercise any unfavorable influence on the health of the workmen engaged in it, but has in some cases a preservative action. We must, however, say, that the facts related to prove this latter proposition are but of little value, and may be reduced to the following. At Morlaix, where a dysentery existed epidemically for two months, few of the tobacco workmen were affected with it, and those in whom it broke out were men of constitutions injured by excesses. At Lyons, where typhus is common, it is rare in those engaged in the manufactory; at Tonneins, where malaria prevailed almost generally, the workmen were exempt from it; lastly, if we may believe M. Siméon, phthisis itself is less frequent, and less rapid in its progress, among this class of people than among the rest of the population.

Before adopting these conclusions, there is a very important point to decide, whether the people employed in the tobacco manufactories are not in a much better condition as regards regularity, work, and wages, than the greater part of the rest of the population. This, in our opinion, is the true cause of the superiority of the classes employed by Government. When the other classes are placed in analogous conditions, analogous results will be obtained.—*Ibid.*

